# From Trials to Practice: Implementing a Clinical Intervention in Community Settings

**DOI:** 10.1177/21501319251339190

**Published:** 2025-05-26

**Authors:** Elizabeth M. Vaughan, Xiaoying Yu, Victor J. Cardenas, Craig A. Johnston, Salim S. Virani, Ashok Balasubramanyam, Christie M. Ballantyne, Aanand D. Naik

**Affiliations:** 1 University of Texas Medical Branch, Galveston, USA; 2 Baylor College of Medicine, Houston, TX, USA; 3 University of Houston, TX, USA; 4 The Aga Khan University, Karachi, Pakistan; 5 University of Texas School of Public Health, Houston, USA; 6 Michael E. DeBakey VA, Houston, TX, USA

**Keywords:** diabetes, patient navigators or community health workers, community clinic, diabetes group visits or shared medical appointments, Hispanic or Latino(a), telehealth, implementation

## Abstract

**Introduction/Objectives:**

Diabetes increases the risk of complications, especially for vulnerable populations. Our previous randomized clinical trial (RCT), TIME (Telehealth-supported, Integrated Community Health Workers (CHWs), Medication access, group visit Education), showed the efficacy of CHW-led diabetes care. This study aimed to gather data on transitioning TIME from clinical trials to practical implementation.

**Methods:**

We conducted a 12-month RCT at a nonprofit community clinic using the Consolidated Framework for Implementation Research (CFIR). Participants, Hispanic adults without insurance and with type 2 diabetes (N = 58; 29/arm), were randomized to TIME (intervention) or usual care (control). The intervention included monthly group visits and weekly CHW mHealth contact (6 months, Action Phase), followed by quarterly visits and bi-monthly mHealth contact (6 months, Maintenance Phase). The research team provided tele-mentoring to the clinic team throughout the intervention. Outcomes included implementation measures including acceptability, adoption, appropriateness, cost, feasibility, fidelity, satisfaction, and effectiveness.

**Key Results:**

The program showed high levels of fidelity (direct observation), adoption (CHW-participant contact: 844 successes of 957 attempts [88.2%]), and feasibility (3.4% attrition). The intervention’s net savings was $16,435 ($566/participant). At 6 months, intervention participants had greater HbA1c reductions (−0.85% vs 0.35% [δ = 1.2%]; *P* = .004; effectiveness) compared to the control. At month 12, more intervention participants improved HbA1c (−0.52% vs 0.25% [δ = 0.8%], *P* = .062) and preventive care adherence (*P* < .0001) compared to the control. Surveys revealed high appropriateness (mean = 4.8/5.0 and 5.95/6.0), satisfaction (mean = 4.6/5.0), and acceptability (mean = 4.9/5.0) among providers, CHWs, participants, and stakeholders.

**Conclusions:**

TIME met key early implementation measures, including strong engagement at both clinic and participant levels, while demonstrating cost savings and significant clinical improvements. These results support the transition of TIME from efficacy trials to practical, community-based diabetes care. Larger studies are needed to further evaluate these findings.

## Introduction

Currently, 537 million adults worldwide are living with diabetes, representing 10% of the global population.^1^ Diabetes is the leading cause of lower extremity amputations, blindness, and end-stage renal disease.^2-4^ It is a condition disproportionately affecting individuals in low- and middle-income populations both in prevalence and healthcare expenditures, with more than 90% of those effected from resource-limited settings, many of whom are under or uninsured.^1,5-8^ Among ethnic groups, Hispanics exhibit one of the highest rates of diabetes prevalence, compounded by factors such as language and cultural barriers, which further elevate the risk of adverse disease outcomes.^8-11^ Addressing these inequities requires innovative and sustainable interventions in practical settings.

Investigators have utilized diabetes group visits—shared medical appointments where individuals receive education, social support, and a provider encounter—to enhance diabetes care.^12^ These programs, typically led by medical providers or ambulatory staff, have been shown to improve glucose control, blood pressure in participants, and self-management skills.^13^ Our research team demonstrated the effectiveness of novel Community Health Worker (CHW)-led diabetes group visits in a randomized clinical trial (RCT), TIME (Telehealth-supported, Integrated CHWs, Medication access, diabetes Education).^14^ This trial led to significant improvements in HbA1c, blood pressure, and weight, with results sustained at 2 years.^15^ Further implementation studies are now essential to transition from efficacy trials to practical applications, collecting data needed for scalability and long-term sustainability.

Implementation is a systematic process aimed at integrating an innovation into routine practice and is essential for ensuring that desired outcomes are achieved.^16^ Proctor et al^16^ classified implementation research into 3 stages. Stage One focuses on implementation outcomes including acceptability, adoption, appropriateness, costs, feasibility, fidelity, penetration, sustainability. Many of these outcomes are linked to early stages of adoption, though penetration, sustainability, and costs may be more relevant during mid to late adoption. Stage Two encompasses service outcomes: efficiency, safety, effectiveness, equity, patient-centeredness, timeliness. Stage 3 includes client outcomes, including satisfaction, function, symptomatology.^16^

This study aimed to collect early stage practical implementation data for the TIME intervention in a practical setting. Our research team tele-mentored a clinic team to implement TIME in their clinic. Participants were low-income, uninsured Hispanics with type 2 diabetes, randomized to either TIME (intervention) or usual care in the clinic (control). We gathered implementation data essential for scalability and sustainability using Proctor’s measures, assessing acceptability, adoption, appropriateness, cost, feasibility, fidelity (Stage 1) and explored key variables for Stage 2 (effectiveness) and Stage 3 (satisfaction).^12^

## Methods

### Setting

We conducted this RCT at a non-federally funded, nonprofit community clinic in Houston, TX. The clinic primarily served Hispanic adults, many of whom have an undocumented status. Eligibility for clinic services required being uninsured and having an annual income at or below 150% of the federal poverty level. The clinic was selected based on patient socio-demographics and data availability to meet the study’s objectives.^17^ The study was approved by Baylor College of Medicine and the University of Texas Medical Branch Institutional Review Board (#49-672, #22-0224) and all participants provided informed consent.

### Theoretical Framework

We applied the Consolidated Framework for Implementation Research (CFIR) as the guiding framework for implementaion.^18^ CFIR contains 5 domains—innovation, outer setting, inner setting, individuals characteristics, and implementation processes—each comprising of 4 to 12 constructs that may be adapted to reflect project-specific language.^18^ CFIR informed the key structural and procedural elements of our intervention within the context of a practical setting. The project-specific terms for each domain were defined as follows: innovation (CHW-led group visits), outer setting (community), inner setting (clinic), individuals (clinic administrators, board; clinic team; implementation leads [research team]; recipients [participants]), and processes (mentored approach, telehealth-support).

### Research and Clinic Teams

The *clinic team* included 9 providers (2 physicians and 7 advanced practice providers) and 6 bilingual (Spanish/English) community members interested in becoming certified CHWs. The *research team* comprised of 2 primary care study physicians and 6 CHW instructors (CHW-Is) certified in Texas, a certification requiring 160 h of state-approved coursework.^19^ CHW-Is averaged 8 years of experience leading community diabetes programs on our team. The research team, including CHW-Is, completed IRB-approved consent training. We established a nonprofit organization (Mi Promotor de Salud) that served as a Texas-approved CHW certification program (#99).^20^

#### Clinic Team Training

This study used telehealth, defined as the delivery of health information via virtual platforms, rather than telemedicine, which involves practicing medicine remotely.^21^ Before the study, the clinic providers did not have experience with group visits. Research physicians provided a 1-h, telehealth introductory session, followed by a 3-h virtual training on facilitating group visits and conducting patient encounters.^22^

CHW training was conducted via telehealth and is detailed elsewhere.^23-25^ To accommodate work schedules, training sessions were held after hours over 16 months and in 2 parts. First, CHWs completed a 4-month, 160-h state certification course, including weekly 2-h classes (40 h total) and 6 h of weekly interactive homework via text messaging (120 h total). This fulfilled certification course requirements while offering practical activities. Next, CHWs completed a 35-h diabetes course (2-3 h/month) covering self-management topics including medications, lab values, nutrition, exercise, preventive care, coaching strategies, teaching techniques, and patient confidentiality (Health Insurance Portability and Accountability Act [HIPAA]). CHWs completed 17 of the 35 h before the intervention (5 h on teaching fundamentals and 12 h on diabetes essentials), and finished the remaining 18 h during the 12-month intervention for hands-on learning. On average, CHWs worked 3 h per week during the study.

#### Stakeholder Engagement

Six months before the study began, the research team initiated monthly meetings with stakeholders, including the clinic CEO, executive director, medical director, clinic director, nursing supervisor, and volunteer coordinators, to support planning and engagement. The research team provided regular updates on the project’s CHWs, providers, participants, and clinical outcomes.

### Participants

#### Eligibility Criteria

Adults ≥18 years who were Hispanic, Spanish-speaking, and diagnosed with type 2 diabetes (eg, provider documentation, HbA1c ≥6.5%, fasting glucose >125 mg/dL, and/or prescribed oral glucose-lowering medications) were eligible.^26^ Exclusion criteria included Type 1 diabetes, pregnancy, failure to attend at least 1 group visit (intervention) or clinic appointment (control) within the 12-month study, and conditions or medications altering HbA1c values (eg, blood transfusions or alternating steroid doses).^27^ Eligible individuals were identified through a database using codes for Hispanic/Latino(a) and type 2 diabetes (ICD 10 E11.9).

### Intervention

Our prior studies provided the structure of the intervention.^14,23,28^ Participants were randomized to CHW-led group visits (intervention) or usual care (control). Usual care included routine provider visits (averaging 4/year) and access to nutrition education, food assistance, and pastoral care. To minimize the risk of provider crossover or contamination,^29^ control participants were blocked from the study providers’ schedules. Consistent with prior studies, no contamination effects were observed.^13,14,30^

The intervention included 2, 6 month phases, *Action* (months 1-6) and *Maintenance* (months 7-12). We developed the curriculum using evidence-based literature and our prior studies. It covered core self-management topics, such as nutrition, exercise, and general healthy living, that is, alcohol, smoking, preventive care, sexuality and chronic disease, and depression.^14,23,28^ During the Action Phase, participants attended monthly sessions (6 visits total), and during the Maintenance Phase, they attended quarterly sessions (2 visits total). Each group visit included CHW-led diabetes education in a large with small group breakout sessions, an individual provider encounter for medication adjustment, and a healthy meal or snack. After each group visit, CHWs and providers met to discuss any patient issues or updates.

Between group visits, CHWs contacted participants weekly (months 1-6) and semi-monthly (months 7-12) via mHealth (text or phone). They provided coaching on diabetes self-management, including glucose monitoring, medication management, body mass index (BMI), weight loss, physical activity, and clinic navigation. Key points were recorded on a secure online spreadsheet, and issues were addressed weekly by a CHW representative with a designated clinic provider.

### Measures

Table 1 details implementation outcomes by Stage. For cost outcomes, we referenced prior investigators who found that for each 1% reduction in HbA1c, individual diabetes-related costs were reduced by 13% ($736) per year.^31^

**Table 1.** Implementation Outcomes by Implementation Stage.^16^

Measure (implementation stage)	Definition, description, and outcomes
Acceptability (Stage 1)	Definition: stakeholder satisfaction Outcomes: 15-question stakeholder satisfaction survey at 12 months using our prior work as templates (n = 12 questions on Likert scale and n = 3 open-ended questions)^14,15,28^
Adoption (Stage 1)	Definition: utilization of the intervention Outcome: success defined the ability for CHWs to contact patients through mHealth (mobile health)
Appropriateness (Stage 1)	Definition: perceived fit with the clinic Outcomes: 24–question Telehealth Usability Questionnaire (n = 21 questions on Likert scale and n = 3 open-ended) at month 12^32^ (providers) and 12-question Texas Department of State Health Services CHW evaluation at 12 months (n = 9 questions on Likert scale and n = 3 open-ended; CHWs)^19^
Cost (Stage 1)	Definition: healthcare costs associated with the intervention and usual care Outcomes: costs associated with mean HbA1c change^a^
Effectiveness (Stage 2, exploratory)	Definition: Clinical outcomes Outcomes: Change of HbA1c, blood pressure, weight body mass index (BMI) from baseline to 6 and 12 months; achievement of HbA1c control (<7% if <64 years and <7.5% if ≥65 years) at 6 and 12 months; Change of lipid levels from baseline to 12 months; Achievement of 6 American Diabetes Association preventive care measures at 12 months (screening for B12 deficiency, urine microalbumin, diabetes retinopathy; received influenza vaccination, diabetes foot exam, statin as clinical indicated)
Feasibility (Stage 1)	Definition: practicability of the intervention Outcome: success defined as <20% attrition^33,34^
Fidelity (Stage 1)	Definition: intervention carryied out as intended Description: *Providers*: had brief (15-30 min) meetings at group visits prior to seeing participants; research physician was available during the group visit* and held 2-h telehealth office hours weekly *Community Health Workers (CHWs)*: CHW-instructors (CHW-Is) were available during the group visits^b^. CHW-Is met 1:1 with CHWs for mentoring and with the group weekly via telehealth for support and training. Outcomes: direct observation of (1) CHWs and providers performing job duties and (2) progress notes in electronic medical record (provider) and secure online spreadsheet (CHWs)
Participant satisfaction (Stage 3, exploratory)	Definition: Participant satisfaction Outcomes: 10-question participant survey at 12 months using our prior work as templates (n = 7 questions on a Likert scale and n = 3 open-ended).^14,15,28^

a Each 1% reduction in HbA1c is associated with a 13% reduction in diabetes-related costs per patient per year ($736).^31^

b Occurred in-person for the first 2 group visits and via telehealth thereafter.

### Statistical Analysis

Effectiveness (clinical outcomes) analyses were performed using SAS 9.4 (SAS Institute, Inc.). All tests were 2-sided with a significance level of *P* < .05. Descriptive statistics, such as mean and standard deviations for continuous variables and count and proportions for categorical variables, were calculated by groups at baseline and follow up time points. For continuous variables, changes were derived for month 6 and month 12 outcomes. In cases where HbA1c levels were not available but glucose levels were (n = 4 at month 6, n = 0 at month 12), we converted these values to HbA1c using the conversion equation ([46.7 + mean glucose]/26.7), applying statistical procedures.^15,35^ This approach utilizes all available data, enhancing statistical power, particularly in small sample sizes, making it a more robust and effective method. To complement between-group comparisons (TIME vs usual care), we also conducted within-group analyses to assess changes over time, enhancing the sensitivity to the intervention’s effects.

Missing data patterns and distributions for the outcomes were examined prior to analysis. Given low attrition and the robustness of non-parametric tests, changes within each group and between arms were compared using Wilcoxon signed rank test and Wilcoxon rank sum test, respectively, based on observed data. To examine the impact of missing data (6 months: n= 10 HbA1c; n = 11 weight; n = 12 blood pressure; 12 months: n = 3 HbA1c, n = 4 weight, n = 6 blood pressure; and n = 4 no cholesterol values), we applied linear mixed models, which assumes missing at random given observed outcomes and covariates. The model included fixed effects of group, time and group by time interaction, and random intercept to account for the correlation among the repeated measures from the same subject. It also addressed a higher amount of missing data at 6 months, primarily in the control arm as it assumes data are missing at random, similar to the assumption used in multiple imputation (MI), though MI explicitly imputes missing values. For instance, under the mixed model, we assumed that the missing A1c value at month 6 depended on whether the individual was in the intervention or control group, as well as on the observed A1c values at baseline and month 12. In contrast, relying only on complete data at month 12 would assume data were missing completely at random, meaning that missing A1c values were unrelated to baseline or month 6 value, was less plausible. The mixed model also included all available data, which increased statistical power compared to a complete case analysis that only uses data from 2 time points.

For the repeatedly measured secondary binary outcome, generalized estimating equation (GEE) was used. The mixed model analyzed HbA1c as a continuous variable and compared changes in HbA1c values between the intervention and control groups, which was our primary analysis. To address the clinically important question of whether HbA1c improvement differed between groups in patients with uncontrolled diabetes, we defined a binary outcome (improvement vs no improvement) at each time point and used a GEE model to analyze repeated binary measures (improvement at 6 and 12 months) and assessed the significance of the intervention effect at each time.

## Results

**Figure 1.**
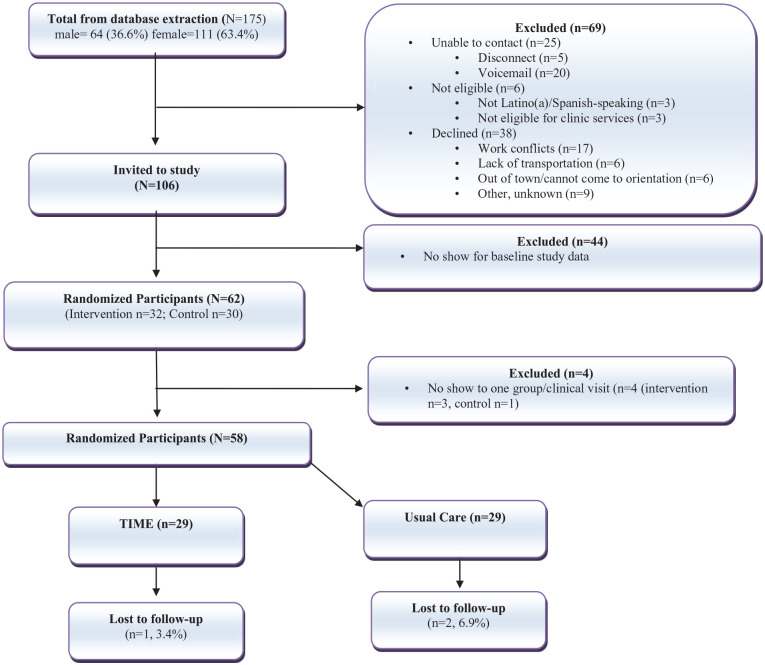
CONSORT diagram of low-income Latino(a)s participating in the TIME (Telehealth-supported, Integrated Community Health Workers, and Medication-access, Education in group visit) program.

The CONSORT diagram (Figure 1) shows participant flow through the intervention and control arms, from recruitment to randomization and loss to follow-up. Of 175 contacted individuals, 63.4% were female. Sixty-nine were excluded: 25 could not be reached, 6 were ineligible, and 38 declined, mainly due to scheduling conflicts (76.3%). Of the 106 who expressed interest, 44 did not attend baseline data collection, leaving 62 participants randomized to the intervention (n = 32) or control (n = 30). Four consented participants missed all group visits or clinic appointments and were excluded, resulting in 58 participants in the final analysis (29/arm).

Baseline demographics of participants are detailed in Table 2. Most participants were employed in domestic or manual labor positions. The majority received oral glucose-lowering medications, nearly half had hypertension, and fewer than 10% had a history of coronary artery disease, cerebrovascular accident, or chronic kidney disease.

**Table 2.** Baseline Demographics, n = 58 (Intervention, n = 29; Control, n = 29).

	Intervention	Control
Variable	mean (±SD)	mean (±SD)
Age (years)	52.1 (7.1)	53.5 (8.1)
	n (%)	n (%)
Sex		
Female	13 (44.8)	19 (65.5)
Male	16 (55.2)	10 (34.5)
Legal documentation		
Known status	1 (3.5)	1 (3.5)
Unknown/undocumented status	28 (96.5)	28 (96.5)
Employment		
Manual labor	10 (34.5)	6 (20.7)
Domestic	7 (24.1)	10 (34.5)
Administration	2 (6.9)	1 (3.4)
Food service	2 (6.9)	2 (6.9)
Unemployed	5 (17.2)	6 (20.7)
Other/unknown	3 (10.3)	4 (13.8)
Diabetes therapy		
Lifestyle only	0 (0.0)	1 (3.4)
Oral medications only	22 (75.9)	27 (93.1)
Injectables only	2 (6.9)	0 (0.0)
Oral + injectables	5 (17.2)	1 (3.4)
Receiving PAP medication	9 (28.1)	9 (31.0)
Past medical history		
Hypertension	12 (41.4)	16 (55.2)
Coronary artery disease	0 (0.0)	0 (0.0)
Cerebrovascular accident	2 (6.9)	0 (0.0)
Chronic kidney disease	1 (3.4)	2 (6.9)

Abbreviation: PAP, prescription assistance program.

Retention rates were 96.6% in the intervention group and 93.1% in the control group. Average class attendance for the intervention was 65.4% (range 46.7%-83%, Phase 1: 69.4%, Phase 2: 53.3%), with a mean of 5 visits per participant and 83% (n = 24) attending at least half of the classes. Attendance was highest in Month 1 (n = 25) and lowest in months 9 (n = 14). At 12 months, 8 participants lost clinic eligibility (5 from the intervention group, 3 from the control) due to insurance changes (n = 7) or deportation (n = 1). The clinic ensured all participants could receive 12-month laboratory tests, regardless of eligibility status.

### Acceptability

Stakeholder satisfaction was very high, with an average score of 4.9/5.0 (range = 4.6-5.0) on a 15-item survey. Stakeholders appreciated increased patient engagement, a diverse range of educational topics, enhanced trust through the CHW relationship, and improved access to care. Many suggested expanding future programs to address other chronic conditions.

### Adoption

CHWs successfully contacted participants 844 out of 957 times (88.2%; Phase 1: n = 518/609 [85.1%], Phase 2: n = 326/348 [93.7%]).

### Appropriateness

Providers rated the training highly, with an average score of 4.8/5.0 (range = 3.7-5.0) on the 21-item Telehealth Usability Questionnaire. Open-ended feedback emphasized the value of the training materials and a sense of contributing to participants’ health improvement. CHWs expressed high satisfaction with the training, with an average score of 5.95/6.0 (range = 5.8-6.0) on the 11-item Texas Department of State Health Services survey. CHWs reported that the training equipped them with the skills to assist patients with goal-setting and preventing complications.

### Cost Effectiveness

When applying the mean HbA1c changes in each group (intervention: −0.9%, control: 0.25%), the annual cost savings for the intervention group amounted to $11,099 ($736 per 1-point HbA1c reduction × −0.52% mean HbA1c reduction × 29 participants), while the control group annual loss totaled $5,336 ($736/1-point HbA1c reduction × +0.25% mean HbA1c increase × 29 participants). The net savings was $16 435 or $566 per participant.

### Effectiveness

Table 3 summarizes clinical outcomes for both study arms. At 6 months, 75.0% intervention and 60.0% control participants achieved target HbA1c levels, with a net difference of 10 individuals (*P* = .017). At 12 months, 60.7% intervention and 51.9% control participants met target HbA1c levels, resulting in a net difference of 4 (*P* = .439). More intervention participants improved HbA1c levels at both 6 (86.7% vs 54.6%, *P* = .018) and 12 months (73.3% vs 35.7%, *P* = .051). Weight reduction was more common in intervention participants at 6 months (44.4% vs 38.1%, *P* = .666) and 12 months (50.0% vs 26.9%, *P* = .085) but not significant. The intervention group had superior preventive care outcomes, including higher rates of statin prescriptions (*P* < .001), serum B12 measurement, diabetes foot exams, eye referrals, and urine microalbumin screening (all *P* < .0001). Influenza vaccination rates were similar between groups (*P* = .184).

**Table 3.** Clinical Outcomes of the Intervention and Control Groups (N = 58; 29/Group).

Variable	TIME (intervention)					Usual care (control)					*P*-value	
	Baseline	Month 6	Month 6 net change	Month 12	Month 12 net change	Baseline	Month 6	Month 6 net change	Month 12	Month 12 net change	Month 6^a^	Month 12^a^
	n (%)					n (%)						
Metrics												
Target HbA1c^b^	13 (44.8)	21 (75.0)	8	17 (60.7)	4	14 (48.3)	12 (60.0)	−2	14 (51.9)	0	.017	.439
Any HbA1c improvement^c^	—	13 (86.7)	—	11 (73.3)	—		6 (54.6)		5 (35.7)		.018	.051
Any weight improvement^d^	—	12 (44.4)	—	14 (50.0)	—		8 (38.1)		7 (26.9)		.666	.085
Preventive Care												
Statin	—	—	—	29 (100)	—	—	—	—	20 (69.0)	—	—	<.001
B12 (serum)	—	—	—	29 (100)	—	—	—	—	2 (6.9)	—	—	<.0001
Diabetes foot exam	—	—	—	29 (100)	—	—	—	—	4 (13.8)	—	—	<.0001
Eye referral	—	—	—	29 (100)	—	—	—	—	14 (48.3)	—	—	<.0001
Urine microalbumin	—	—	—	29 (100)	—	—	—	—	10 (34.5)	—	—	<.0001
Influenza vaccine^e^	—	—	—	22 (81.5)	—	—	—	—	19 (65.5)	—	—	.184
	Mean (±SD)					Mean (±SD)						
HbA1c (%)												
All individuals	7.98 (2.17)	6.96 (1.54)	−0.85 (1.58)^g^	7.38 (1.83)	−0.52 (1.54)	7.58 (1.75)	7.96 (1.53)	0.35 (1.53)	7.86 (2.31)	0.25 (2.66)	.004	.062
Uncontrolled^f^	9.39 (1.95)	7.73 (1.76)	−1.45 (1.93)^g^	8.44 (1.89)	−0.90 (2.00)	8.56 (1.83)	8.78 (1.00)	0.25 (1.66)	8.88 (2.75)	0.19 (3.68)	.015	.174
Blood Pressure (mmHg)												
Systolic	129.3 (18.1)	131.5 (17.1)	2.4 (11. 6)	127.2 (14.8)	−3.1 (15.1)	130.2 (14.3)	127.2 (14.8)	−2. 7 (17.0)	131.1 (21.6)	1.6 (20.2)	.217	.408
Diastolic	80.6 (6.6)	79.4 (5.6)	−1.0 (5.1)	76.9 (6.6)	−3.6 (6.1)**	79.5 (9.3)	76.5 (7.1)	−0.8 (7.6)	77.5 (8.6)	−1.3 (10.4)	.826	.500
Weight (lb)	192.613 (35.511)	194.3 (34.1)	−0.3 (8.9)	194.4 (34. 6)	0.5 (9.0)	181.0 (32.4)	175.4(37.3)	1.7 (5.1)	185.7 (38.7)	4.3 (9.0)**	.312	.170
BMI (lb/ft^2^)	33.112 (5.7)	33.311 (5.2)	−0.08 (1.5)	33.4 (5.3)	0.06 (1.6)	31.9 (5.7)	30.9 (6.0)	0.24 (0.9)	32.7 (6.8)	0.7 (1.6)	.354	.145
Cholesterol												
Total	147.5 (35.5)	—	—	164.2 (42.8)	15.6 (47.2)	180.4 (42.3)	—	—	195.7 (46.5)	14. 5 (44.4)	—	.951
LDL	90.8 (30.7)	—	—	87.3 (32.3)	−1.4 (37.6)	105.5 (32.1)	—	—	111.3 (29.8)	4.9 (28.4)	—	.910
HDL	34.8 (11.1)	—	—	44.0 (9.7)	9.5 (9.8)***	40.0 (10.9)	—	—	47.1 (12.3)	7.4 (8.9)***	—	.207
Triglycerides	167.1 (120.2)	—	—	210.6 (133.5)	36.7 (127.1)	160.1 (125.7)	—	—	198.6 (108.0)	38.2 (151.5)^e^	—	.498

a Between group analyses comparing intervention vs control arms changes from baseline to 6 or 12 months.

b A1c <7% if <64 years old or <7.5% if ≥65 years old. Six and 12-month denominator reflects those with 6 and/or 12-month data (6 months: intervention n = 28, control n = 20; 12-months intervention n = 28, control = 27) and change denominator reflects the total n/arm (n = 29/arm).

c Denominator includes any improvement in HbA1c levels for individuals not at target at baseline and who have data at 6 and/or 12 months (6 months intervention n = 15, control n = 11; 12 months intervention n = 15, control n = 14).

d Six and 12-month denominator reflects those with 6 and/or 12-month data (6 months: intervention n = 27, control n = 21; 12-months intervention n = 28, control = 26).

e Intervention n = 27 due to n = 2 with allergic response/vaccine intolerance;

f Within group (intervention vs intervention or control vs control) significance *P* < .05.

g HbA1c ≥7% if <64 years old or ≥7.5% if ≥65 years old at baseline; within group significance of ** *P* < .01, *** *P* < .001.

Glycemic control improved in the intervention group and worsened in the control group. The intervention group reduced HbA1c by −0.85% (SD = 1.58) at 6 months while the control group showed increases in HbA1c of 0.35% (SD = 1.53; δ = 1.2%, *P* = .004). Intervention participants also reduced HbA1c levels at 12 months by −0.52% (SD = 1.54) at 12 months while the control arm increased 0.25% (SD = 2.66; δ = 0.8%, *P* = .062). The control group showed weight and BMI increases, with a 4.3-pound weight gain at 12 months though differences between groups were not significant. Blood pressure and lipid outcomes were similar between arms. A linear mixed model with missing data adjustments confirmed these results.

GEE analyses showed significantly higher odds of HbA1c improvement in the intervention group at both 6 and 12 months (OR = 4.65; 95% CI = 1.3, 17.6, *P* = .02). A significant interaction between groups over time was observed (*P* = .01), with a difference at 6 months (*P* = .001) but not at 12 months (*P* = .27). From baseline to 6 months, the intervention group had 3.4 times higher odds of controlled glycemia (95% CI = 1.61-7.26, *P* = .001), while the control group showed no significant change (OR = 0.74, 95% CI = 0.42-1.29, *P* = .28). At 12 months, the intervention group had marginally higher odds of controlled glycemia (OR = 1.8, 95% CI = 0.92-3.40, *P* = .09), with no significant change in the control group (OR = 1.02, 95% CI = 0.51-2.06, *P* = .95). At 6 months, intervention participants were 4.7 times more likely to have better controlled glycemia than controls (95% CI = 1.82-11.9, *P* = .001), with a non-significant 1.7-times improvement at 12 months (95% CI = 0.66-4.50, *P* = .27).

### Feasibility

The intervention demonstrated high practicability, as shown by the low attrition rate of 3.4% among intervention participants.

### Fidelity

Fidelity was assessed through program adherence. Direct observation of provider-participant interactions and electronic medical record (EMR) reviews confirmed that providers followed training algorithms and prescribed affordable, accessible medications. Observations of CHW education sessions indicated adherence to the intended curriculum in both small and large groups, with high-quality, detailed notes maintained during their weekly mHealth interactions with participants.

### Participant Satisfaction

Participants rated their satisfaction an average of 4.6/5.0 (range = 3.9-5.0). They particularly valued the personalized attention, education, and the support from CHWs and providers. Suggestions for improvement included offering classes at different times or days and expanding the clinic’s services to other locations.

## Discussion and Conclusions

Innovative and sustainable interventions are essential for reducing complications in economically marginalized populations. In this study, we assessed the transition of our model, TIME, previously shown to be effective in a tightly controlled randomized clinical trial to a practical setting. We found that a telementoring approach successfully met key implementation outcomes, providing data necessary for moving from clinical trials to practical, community-based effectiveness.

The intervention demonstrated positive implementation outcomes. It showed both cost and clinical effectiveness, with net savings of $566 per participant and $16 435 for this study of 29 intervention participants, along with improved glycemic control, weight, and preventive care. Larger studies would likely amplify cost-savings. GEE analyses showed significantly higher odds of improved glycemic control at 6 and 12 months, also indicating potential for better outcomes and reduced healthcare costs. The greater reduction in HbA1c observed in the intervention group during the more intensive Action Phase (monthly group classes and weekly CHW communication) may be attributed to increased exposure. However, lower attendance coupled with higher CHW-participant contact rates in the Maintenance Phase suggests that exposure alone may not fully account for the clinical outcomes. In our previous studies, we found that initial motivation often declined over time, which could contribute to the larger initial HbA1c reductions.^14,15,28^ Nonetheless, the Maintenance Phase, with less frequent exposure, likely better reflects practical implementation, and the sustained HbA1c improvements alongside effective CHW-participant interactions observed in this phase were promising.

The intervention also demonstrated significant improvements in preventive care outcomes relative to the control group. The exact mechanisms underlying these findings are likely multifactorial, with potential contributions from provider training from the research team, inclusion of group visits for comprehensive care, and the ongoing reminders provided by CHWs to participants throughout the study. Further, surveys showed high program acceptability and satisfaction among CHWs, providers, participants, and stakeholders, with participants valuing personalized care and providers appreciating training materials. CHWs had strong adoption, contacting participants 88.2% of the time, and feasibility was shown by low (3.4%) attrition. Fidelity was maintained through observations and EMR reviews, ensuring protocol adherence and effective medication prescribing.

Several barriers were addressed and facilitators incorporated to support this transition. Risk of inadequate support was mitigated by ongoing telementoring for CHWs and providers, which reduced clinic and research team burden and enabled the work to continue without logistical challenges. Although clinic providers had primary care experience, managing diabetes pharmacotherapies in low-income settings presents unique challenges. With only 3 of 12 glucose-lowering medications available at affordable prices (<$10/month) and complex Prescription Assistance Program applications for others,^36^ provider-to-provider mentorship was crucial to navigate these processes.

Similarly, mentorship provided critical assistance for CHWs, who often work in the field with inadequate support. Studies have shown that CHWs welcome support; 73% of CHWs viewed team meetings as essential to successful implementation.^37^ A qualitative analysis found that CHWs struggled with setting boundaries, particularly when ending communication after program completion^38^—a barrier often overlooked in the literature. Ongoing support beyond initial training is vital for successful implementation. We observed that as CHWs worked with participants, they frequently had questions about applying their training, and consistent mentorship helped them find answers, reducing the risk of misinformation.

Facilitating stakeholder buy-in was key to successful implementation. This was achieved through consistent communication at all levels, including monthly research team-stakeholder meetings that addressed issues and logistical concerns before they became significant challenges. The integration of CHWs and providers during group visits emphasized the value of collaboration between these typically parallel roles. For instance, providers used this time to highlight medication adherence concerns, while CHWs relayed participant barriers such as cost or eligibility. They then collectively incorporated strategies to resolve issues.

This study has several strengths. We addressed key facilitators to overcome barriers related to support, communication, and logistical, and used a detailed methodology to describe the implementation processes, providing a valuable template for community-based implementation work.^39^ We also introduced a novel telementoring approach for both CHWs and providers. However, generalizability may be limited by the focus on a Hispanic population at a single clinical site.

This study highlights the successful transition of an efficacious clinical trial into a practical, community-based program. Implementation measures and exploratory outcomes showed promising trends, suggesting that the potential impact of telementoring in facilitating accessible mentoring and improving implementation interventions. Future research is needed to evaluate later stages of implementation including a robust analysis of intervention and usual care costs and further expand on these findings in larger trials.
